# Effectiveness of high intensity and sprint interval training on metabolic biomarkers, body composition, and physical fitness in adolescents: randomized controlled trial

**DOI:** 10.3389/fpubh.2024.1425191

**Published:** 2024-07-31

**Authors:** Noelia González-Gálvez, José Francisco López-Gil, Alejandro Espeso-Garcia, Lucia Abenza-Cano, Adrián Mateo-Orcajada, Raquel Vaquero-Cristóbal

**Affiliations:** ^1^Facultad del Deporte, UCAM Universidad Católica de Murcia, Murcia, Spain; ^2^One Health Research Group, Universidad de Las Américas, Quito, Ecuador; ^3^Faculty of Sport Sciences, Research Group Movement Sciences and Sport (MS&SPORT), Department of Physical Activity and Sport, University of Murcia, San Javier, Spain

**Keywords:** physical activity, exercise, blood pressure, HIIT, SIT, cardiovascular

## Abstract

**Objective:**

The aim of this study was to evaluate the effect of HIIT and SIT programmes on body composition, blood pressure, lipid profile, glucose, cardiorespiratory fitness, and strength of adolescents and to compare the effect between those different protocols.

**Methods:**

Sixty adolescents were recruited from a high school and were randomly placed into three groups. SIT and HIIT undertook a training for 8 weeks, twice a week, for 12 min per session, during their Physical Education lessons. SIT group performed 6 sets of 60 s of work (90-95%HRmax) / 60 s of rest (50-55%HRmax), and HIIT group performed 3 sets of 2 min of work (80-85%HRmax) / 2 min of rest (50-55%HRmax).

**Results:**

After adjustment by sex, both experimental groups exhibited a significant reduction in fat mass (*p* < 0.01), and trunk fat mass (*p* < 0.01), as well as a significant increase in lean mass (*p* = 0.01; <0.01), hand-grip strength (*p* < 0.01) and standing long jump (*p* = 0.05–0.04, respectively). In addition, HIIT showed a significant (*p* < 0.05) improvement in blood pressure, diastolic blood pressure, heart rate and VO2max, and a tendency toward a significant reduction in low density lipoprotein.

**Conclusion:**

The implementation of a HIIT protocol within high school Physical Education sessions, maintained for 8 weeks, at a rate of 3 sets of 2 min of work (80–85% RHR)/2 min of rest (50–55% RHR) generated adaptations such as improved fitness condition, changes in body composition, and improvements in blood parameters and blood pressure. However, the group of adolescents who performed SIT, shorter but more intense sets, did not experience as many benefits.

## 1 Introduction

Cardiovascular diseases remain the foremost cause of mortality in developed countries, highlighting the critical need for early detection and intervention as preventive measures, commencing from early stages of life (1). Early initiation of prevention strategies is imperative during childhood, as research has identified several risk factors associated with the development of cardiovascular diseases in adulthood, including high blood pressure, elevated triglyceride and total cholesterol levels, low levels of high-density lipoprotein (HDL), increased insulin resistance, and high waist circumference, all of which can be observed during childhood or adolescence (2). Moreover, cardiovascular diseases are commonly linked with other medical conditions including metabolic syndrome (3). There is a clear consensus on the beneficial effects on health and the prevention of chronic pathologies of systematic physical activity from an early age (4, 5). One of the recognized effects of systematic physical activity is the improvement of physical fitness, with a negative correlation found between physical fitness and cardiovascular and cardiometabolic risk (6).

Because of the healthful effects of consistent physical activity, the World Health Organization (WHO) recommends an average of 60 min of moderate to vigorous physical activity per day for children and adolescents, as well as those that strengthen muscle and bone should be incorporated at least 3 days a week, and indicates that vigorous physical activity provides the most health benefits (4). However, most children and adolescents do not comply with these recommendations (7–9).

In the search to maximize the health effects of the practice of physical activity within the school environment, minimising the time spent, an alternative could be to carry out high intensity interval training (HIIT), as this type of training has been shown to produce similar adaptations to classic aerobic exercise programmes with a shorter duration of the sessions by working at a higher intensity than what has been done classically (8, 10, 11). Not surprisingly, previous studies have found HIIT to be an effective method for inducing healthy improvements in adolescents with overweight or obesity (12) or for the improvement of the physical fitness of adolescents within the sports environment (13) and studies in schoolchildren do not provide conclusive results for all health-related variables (14). This could be due to the low quality of the research designs and the wide variety of intervention protocols applied (7–9, 14). Although there seems to be widespread evidence that HIIT improves cardiorespiratory fitness in the aforementioned areas, there is more debate about its effect on cardiometabolic markers, which could be due to the diversity of training protocols proposed or the differences in the characteristics of the sample included for each study. In addition, these reviews pointed out as limitations the lack of studies analysing the optimal dose to maximize the benefits of this type of training.

In this sense, different protocols have emerged applying higher intensities and shorter durations, called sprint Interval training (SIT) (15, 16). In this sense, Bucchet and Laursen (15) consider as SIT as an effort or short duration (≤ 60 s) with a maximum intensity and as HIIT as a longer effort (>60 s up to 5 min) and an intensity close to the maximum. The manipulation of intensity and duration of effort has been confirmed as elements that determine energy and metabolic stress (17), thus producing different impacts on the organic systems (15). For example, bouts of maximal or supramaximal intensities with short durations should affect the neuromuscular system, while submaximal intensities close to maximum will mainly improve cardiorespiratory and cardiovascular fitness (15).

A systematic review reports that HIIT and SIT protocols generate similar cardiorespiratory fitness gains in adults (18) but different effects on other variables have been reported (19), and their effect on different variables related to cardiovascular health in adolescents has not been investigated. In addition, another limitation of the above-mentioned systematic reviews with meta-analyses addressing this topic is the lack of articles of high quality, with few studies following a randomized clinical trial design (7). The protocols applied to children and adolescents in the school environment are different, with protocols between 60 and 120 s being mainly used (8, 20, 21). The viability of 30-s protocols has been questioned as it has been shown to show worse adherence and negative sentiment (22); likewise, it is necessary to use specialized equipment to achieve adequate intensities (23), while the 1-min protocols have demonstrated high values of enjoyment and satisfaction in adolescents (24). Some recent research has focused one-to two-minute-long submaximal protocols (8, 20, 21). There are few investigations that apply periods longer than 4 min in the school environment; this requires longer session durations, which are not feasible to be applied in the school environment. In this sense, comparing protocols that may be susceptible to use in the school environment is necessary. Therefore, the aim of this study was to evaluate the effect of HIIT and SIT programmes on body composition, blood pressure, lipid profile, glucose, cardiorespiratory fitness, and strength of adolescents and to compare the effect between those different protocols. The hypothesis of the present research was that both intervention programs will produce improvements in all variables, however, their effects will not be the same, with HIIT programs showing superior effects than SIT programs.

## 2 Materials and methods

### 2.1 Participants

A total of 60 adolescents were recruited from a high school located in the Region of Murcia (Spain) and were randomly randomized into three groups: SIT (*n* = 20), HIIT (*n* = 20), and Control Group (CG) (*n* = 20). The inclusion criteria were (a) being in Compulsory Secondary Education; (b) being physically active in physical education sessions; (c) agree to participate in the study and sign the parent/guardian and adolescents consent form; (d) Be present at the time of the assessments; and (e) no changes in out-of-school physical activity during the intervention period. The exclusion criteria were presenting any musculoskeletal, neurological, cardiological, metabolic or rheumatic pathologies prior to the intervention or at any time during the intervention that would prevent the practice of required (a) physical activity and (b) change of school. The sample size and power calculations were performed using Rstudio 3.15.0 software. The significance level was set at *α* = 0.05 and a power of 95% (1-*β* = 0.95). Standard deviation was used according to standard deviation for cardiorespiratory fitness in previous studies (25). To detect the minimum clinically significant change of a total of 4.064 mL/kg/min (26), a total of 30 participants were required. Considering a dropout rate of up to 6%, we aim to enroll at least 32 participants.

### 2.2 Study design

This randomized controlled trial study was conducted in a secondary school in Murcia (Spain). A stratified randomization method with the software Microsoft Excel (version 2016) was used to distribute subjects into SIT, HIIT and Control Group (CG). Three groups were established based on cardiorespiratory fitness, and then a randomized sequence was generated for these three groups using simple randomization. The group assignment was blinded to the examiner and staff who performed the statistical analysis. The trial design was registered with ClinicalTrials.gov (Code: NCT05544370) and followed the Consolidated Standards of Reporting Trials guidelines and Template for Intervention Description and Replication (TIDIER) checklist.

All adolescents and parents/guardians signed an informed consent form. This project was developed with a research grant from Universidad Católica de Murcia Research Projects Program (PMAFI-11/19) and through a contract/agreement with the Ayuntamiento de Archena (CO/AY/58–20). Ethical approval for this study was obtained from Universidad Católica de Murcia (CE061914) and was implemented according to the guidelines for human research of the Helsinki Declaration.

### 2.3 Procedures

In order to develop the protocols, it was used the classification suggested by Bucchet and Laursen (15) that considered as SIT as an effort or short duration (≤ 60 s) with a maximum intensity and as HIIT as a longer effort (>60 s up to 5 min) and an intensity close to the maximum. Both experimental groups developed a training, HIIT or SIT, for 8 weeks twice a week in school Physical Education sessions. The intervention in both groups had a duration of 12 min, using a useful part of the Physical Education sessions. The intervention was applied after a conventional warm-up. The protocol for SIT consisted of 6 sets of 60 s of work [90–95% resting heart rate (RHR)] / 60 s of rest (50–55% RHR), and the protocol for HIIT consisted of 3 sets of 2 min of work (80–85% RHR) / 2 min of rest (50–55% RHR) (Table 1). The work consisted of running. Heart rate (HR) was monitored by means of the PolarTM M430 monitor, encouraging participants to achieve the prescribed heart rate in each protocol. The CG students attended their two regular physical education sessions and did not participate in any specific or structured exercise program that was different from their regular physical education lesson.

**Table 1:** Exercise training data for the SIT (SIT) and HIIT (HIIT) groups.

	Weeks 1–4	Weeks 5–8
SIT (SIT)		
Work-Rest interval duration (s)	60:60	60:60
Work-Rest interval intensity (RHR)	90–50%	95–55%
Number of sets	6	6
Duration (min)	12	12
HIIT (HIIT)		
Work-Rest interval duration (s)	120:120	120:120
Work-Rest interval intensity (RHR)	80–50%	85–55%
Number of sets	3	3
Duration (min)	12	12

SIT, sprint intensity training; HIIT, high intensity interval training; SIT, experimental group1; HIIT, experimental group 2; s, seconds; min, minutes; RHR, reserve heart rate.

### 2.4 Assessment

The same trained researchers measured the variables using standardized conditions. A total of 4 researchers carried out the measurements. The researchers presented more than 10 years of experience in carrying out the tests. Prior to the beginning of data collection, three sessions were held with the researchers to standardize the conditions to carry out the tests. A pilot study was carried out in which 10 subjects were evaluated and all researchers showed good inter- and intra-observer reliability (ICC > 0.80) and excellent, respectively (ICC > 0.90). The laboratory temperature was standardized at 24°C. No warm-up or stretching exercises were performed by the participants before the measurements, and there was a 5-min rest between tests.

Body mass was measured with a portable digital scale (TANITA BF-522 W, Tokyo, Japan), and height was measured with a SECA 217 stadiometer (SECA, Germany). BMI was calculated with the formula: body mass (kg)/stretch stature (m)^2^. Fat mass (FM) (percentage), trunk FM (kg) and lean mass (LM) (kg) were measured with a portable digital scale (TANITA BF-522 W, Tokyo, Japan).

Systolic blood pressure (SBP), diastolic blood pressure (DBP), and pulse rate (PR) were measured with an automatic device (OMRON, model HEM-7113) after 5 min of rest before taking blood pressure. A single device was used to measure all participants pre- and post-test. Measurements were taken on the left arm twice at 5-min intervals, with the participant in a sitting position, using 3 cuffs of different sizes according to arm girth (27, 28). The lowest measures of SBP and DBP were recorded, since the first reading in a series of BP measurements is typically higher when oscillometric devices are employed (29, 30). Mean arterial pressure (MAP) was calculated with the following formula: 1/3 (SBP − DBP) + DBP (27, 28). MBP is used in clinical practice, assessment protocols, and in previous research. This variable allows blood pressure to be used as a single variable (27, 28, 31). Finger stick blood samples were collected between 8:00 and 9:00 a.m. The Afinion™ Analyzer (Alere, Ltd., Stockport, United Kingdom) device was used for the lipid panel test. This test measured total cholesterol (Chol), High-Density Lipoprotein cholesterol (HDL), Low-Density Lipoprotein cholesterol (LDL), Triglycerides (Trig), Non-HDL Lipids (LipidNonHDL) and the Chol/HDL ratio in whole blood, serum and plasma to be used in the diagnosis and treatment of lipid disorders. This device has been used and validated in previous research (31–35). Accutrend sensor was used for glucose test (Accutrend sensor, Roche Diagnostics, Mannheim, Germany). Previous researches have used and validated this device (31, 32, 36).

The Course-Navette test (a 20-m Shuttle run test) was used to assess CRF. This test is a reliable and valid test to assess CRF in this population (37). The participant has to run back and forth 20 m courts, and must cross the 20 m line at the same time that a sound signal is emitted from a pre-recorded tape come with the test kit. The speed is increased by 0.5 km/h each minute from a starting speed of 8.5 km/h. When the participant is no longer able to follow the speed set by the pre-recorded tape, the test stops. The last one half stage completed by the child was noted (28, 37, 38), and the running speed is consulted in the equivalent tables (38). The maximum oxygen consumption (VO2max, mL/kg/min) was estimated from the number of laps performed by the test participants using the equation reported by Leger et al. (VO2max = 31.025 + 3.238 X − 3.248 A + 0.1536 A X; where X = running speed and A = age) (38).

Two tests were used to measure upper and lower muscle strength: Handgrip test and standing long jump test, respectively. A digital grip strength dynamometer was used for performance the handgrip test (TKK 5401; Takei Scientific Instruments Co., Ltd., Tokyo, Japan). Both tests are valid (39) and reliable (39) and have been implemented in this population (40). Handgrip strength was measured in a standing position with the arms at the sides. The participants performed one repetition in each hand to familiarize themselves with the device and the test. Each participant was asked to squeeze the grip with maximal strength for 3 s with the right hand. The highest peak strength (kg) recorded between the three attempts was considered for analysis. Upper lower body strength was measured form a standing position with feet approximately shoulder’s width apart. The adolescent jumped as far forward as possible. The test was performance twice. The longest distance achieved was recorded in centimeters (40).

### 2.5 Statistical analysis

The Kolmogorov–Smirnov test and Mauchly’s *W*-test were used to evaluate the normality and the sphericity of the data. As a result of these tests, parametric tests were performed. The mean and standard deviation were calculated from the quantitative variables, and frequency and percent were used for the qualitative variables.

A two-way ANOVA with repeated measurements and Bonferroni’s correction were used to compare the changes from the baseline between groups, evaluation time interaction and evaluation time. To protect against Type I error, Bonferroni’s correction was used achieve *p* ≤ 0.005 for statistical significance. All analyses were based on intention-to-treat with an error of p ≤ 0.005 established. The statistical analysis was performed using the statistical package SPSS 24.0 for Windows.

## 3 Results

**Figure 1:**
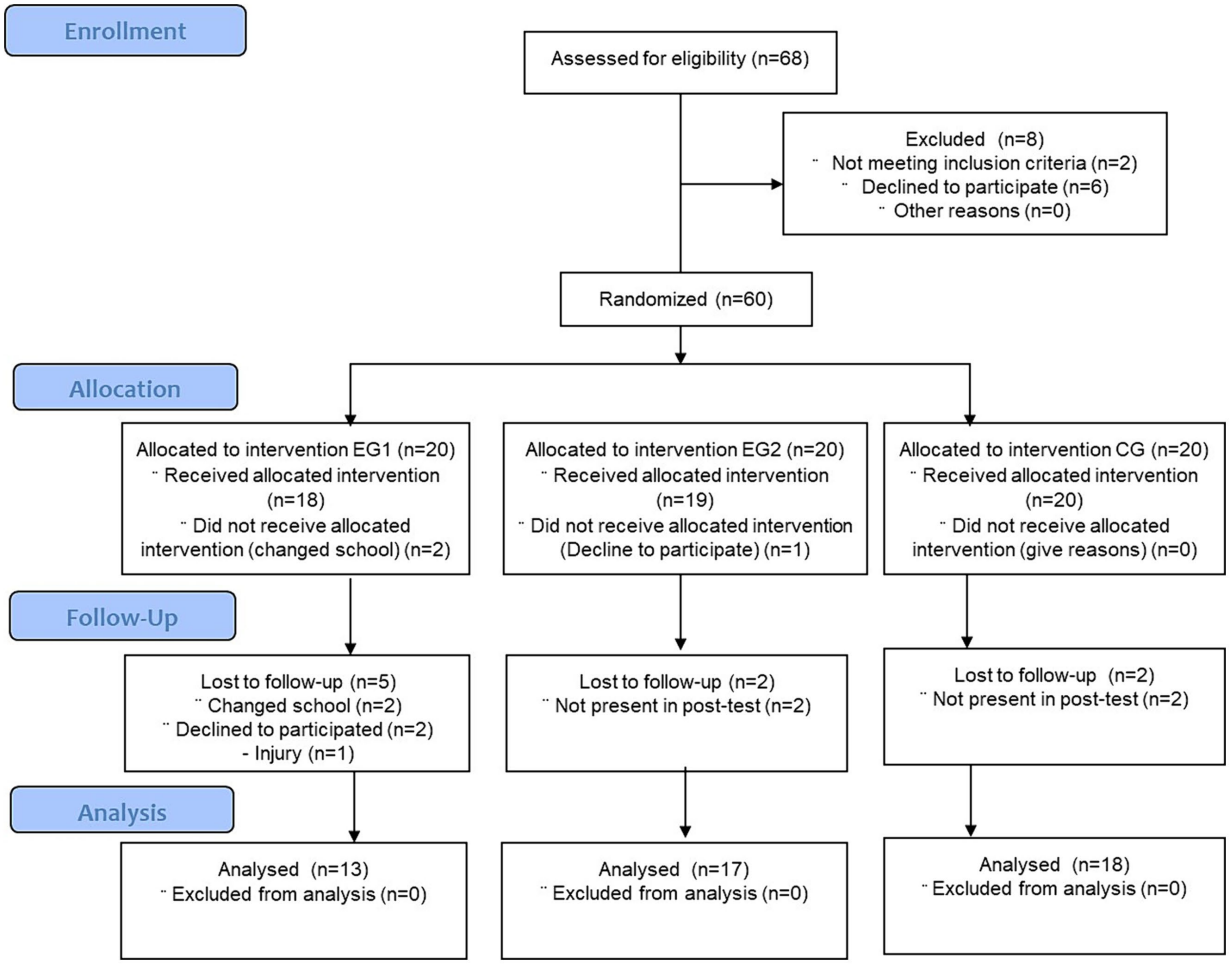
Consolidated Standards of Reporting Trials flow diagram. EG1 = experimental group 1; EG2 = experimental group 2; CG = control group.

Figure 1 shows a flowchart representing the Consolidated Standards of Reporting Trials. The diagram depicts the distribution of participants among the experimental and control groups. SIT, HIIT, and CG comprised 13 (27.08%), 17 (35.42%), and 18 (37.50%) adolescents, respectively. The mean age of the participants was 12.51 ± 0.75 years.

Table 2 presents the unadjusted inter- and intragroup changes for participants who were part of the SIT, HIIT, and CG groups. In addition, it also includes the group*time interaction data for the variables examined in the study. Table 3 shows the same analysis, adjusted for sex. After adjusting for sex, both SIT and HIIT showed a significant reduction in body fat percentage (%), trunk fat mass (kg), and an increase in lean mass (kg), handgrip strength (kg), and long jump (cm). Additionally, the group that performed the HIIT program showed significant improvements in the aforementioned variables, body fat percentage (%), trunk fat mass (kg) and an increase in lean mass (kg), handgrip strength (kg) and long jump (cm), in addition to measurements of blood pressure (BP) (mmHg), heart rate (bpm) and Vo2max (mL/kg/min). Additionally, there was also a trend toward a significant reduction in LDL cholesterol (mg/dL) in this group.

**Table 2:** Differences between groups in the change pre-post intervention and interaction group*time.

Outcome	Group	Pre-test (M ± ED)	Post-test (M ± ED)	No Adjusted			Group * time interaction		
				Difference post-pre (M ± ED)	*p*	95% CI (Mpost-Mpre)	*F*	Sig	ES
BMI (kg/m2)	SIT	22.61 ± 1.25	22.32 ± 1.18	−0.29 ± 0.24	0.23	−0.78; 0.19	0.08	0.92	0.01
	HIIT	20.65 ± 1.08	20.49 ± 1.02	−0.16 ± 0.21	0.44	−0.58; 0.26			
	CG	24.86 ± 1.30	24.68 ± ±1.23	−0.23 ± 0.25	0.37	−0.73; 0.28			
FM (%)	SIT	27.11 ± 2.36	24.67 ± 2.53	−2.44 ± 0.73	<0.01	−3.92; −0.97	5.83	0.01	0.25
	HIIT	22.79 ± ±2.04	18.74 ± 2.19	−4.04 ± 0.63**	<0.01	−5.32; −2.77			
	CG	29.99 ± 2.46	29.31 ± 2.64	−0.682 ± 0.76	0.38	−2.22; 0.86			
Lean mass (kg)	SIT	40.90 ± 2.43	42.08 ± 2.38	1.18 ± 0.44	0.01	0.29; 2.06	5.80	0.01	0.24
	HIIT	38.11 ± 2.10	40.74 ± 2.06	2.63 ± 0.38**	<0.01	1.86; 3.40			
	CG	40.80 ± 2.54	41.56 ± 2.48	0.76 ± 0.46	0.11	−0.17; 1.68			
Trunk FM (kg)	SIT	3.96 ± 0.86	3.38 ± 0.80	−0.58 ± 0.18	<0.01	−0.96; −0.21	1.49	0.24	0.08
	HIIT	3.13 ± 0.74	2.56 ± 0.69	−0.56 ± 0.16	<0.01	−0.88; −0.24			
	CG	4.36 ± 0.90	4.18 ± 0.84	−0.18 ± 0.19	0.35	−0.57; 0.21			
BP (mmHg)	SIT	81.22 ± 3.70	79.94 ± 2.32	−1.28 ± 4.14	0.76	−9.66; 7.10	2.80	0.07	0.13
	HIIT	94.15 ± 3.20	81.02 ± 2.01	−13.13 ± 3.58	<0.01	−20.38; −5.87			
	CG	89.78 ± 3.70	86.47 ± 2.32	−3.31 ± 4.14	0.43	−11.69; 5.07			
SBP (mmHg)	SIT	117.17 ± 5.19	116.83 ± 3.60	−0.33 ± 5.68	0.95	−11.84; 11.17	1.74	0.19	0.09
	HIIT	130.06 ± 4.50	116.44 ± 3.11	−13.63 ± 4.92	0.01	−23.59; −3.66			
	CG	126.00 ± 5.19	122.08 ± 3.60	−3.92 ± 5.68	0.50	−15.42; 7.59			
DBP (mmHg)	SIT	63.25 ± 4.10	61.50 ± 2.54	−1.75 ± 4.37	0.69	−10.60; 7.10	2.33	0.11	0.11
	HIIT	76.19 ± 3.55	63.31 ± 2.20	−12.88 ± 3.78	<0.01	−20.54; −5.21			
	CG	71.67 ± 4.10	68.67 ± 2.54	−3.00 ± 4.37	0.50	−11.85; 5.85			
PR (bpm)	SIT	80.08 ± 4.17	76.08 ± 3.89	−4.00 ± 3.17	0.22	−10.43; 2.43	3.98	0.03	0.18
	HIIT	88.50 ± 3.62	77.06 ± 3.37	−11.44 ± 2.75**	<0.01	−17.01; −5.87			
	CG	78.42 ± 4.17	78.50 ± 3.89	0.08 ± 3.17	0.98	−6.35; 6.51			
Chol (mg/dL)	SIT	162.00 ± 9.95	156.78 ± 9.14	−5.22 ± 3.18	0.12	−11.93;1.49	1.11	0.31	0.06
	HIIT	156.80 ± 9.44	156.20 ± 8.67	−0.60 ± 3.02	0.84	−6.97;5.77			
LDL (mg/dL)	SIT	65.78 ± 8.32	65.44 ± 6.83	−0.33 ± 3.77	0.93	−8.28;7.61	1.65	0.22	0.09
	HIIT	68.90 ± 7.89	61.90 ± 6.48	−7.00 ± 3.57	0.07	−14.54;0.54			
HDL (mg/dL)	SIT	70.22 ± 6.11	69.89 ± 5.38	−0.33 ± 2.55	0.90	−5.71; 5.04	0.06	0.82	<0.01
	HIIT	70.80 ± 5.80	71.30 ± 5.11	0.50 ± 2.42	0.84	−4.60; 5.60			
Trig (mg/dL)	SIT	131.00 ± 13.22	106.67 ± 17.55	−24.33 ± 18.52	0.21	−63.40; 14.73	4.53	0.05	0.21
	HIIT	85.10 ± 12.54	115.10 ± 16.65	30.00 ± 17.57	0.11	−7.06;7.06			
LipidNonHDL (mg/dL)	SIT	92.00 ± 9.09	86.89 ± 8.59	−5.11 ± 2.98	0.10	−11.40; 1.17	0.95	0.34	0.05
	HIIT	86.00 ± 8.62	84.90 ± 8.15	−1.10 ± 2.83	0.70	−7.06; 4.86			
Chol /HDL (mg/dL)	SIT	2.39 ± 0.20	2.33 ± 0.17	−0.06 ± 0.09	0.56	−0.25; 0.14	0.12	0.73	0.01
	HIIT	2.35 ± 0.19	2.25 ± 0.16	−0.10 ± 0.09	0.28	−0.29; 0.09			
Glucose (mg/dL)	SIT	69.22 ± 5.45	60.44 ± 5.43	−8.78 ± 5.89	0.15	−21.21; 3.66	0.12	0.74	0.01
	HIIT	77.00 ± 5.17	71.00 ± 5.15	−6.00 ± 5.59	0.30	−17.80; 5.80			
Vo2máx (mL/kg/min)	SIT	23.50 ± 1.14	24.04 ± 1.23	0.54 ± 0.62	0.39	−0.71; 1.79	3.24	0.051	0.15
	HIIT	23.93 ± 0.88	25.88 ± 0.95	1.943 ± 0.48#	<0.01	0.98; 2.91			
	CG	21.44 ± 0.88	21.77 ± 0.95	0.32 ± 0.48	0.50	−0.64; 1.29			
Handgrip (kg)	SIT	19.93 ± 2.04	23.24 ± 2.32	3.31 ± 0.82	<0.01	1.65; 4.97	4.32	0.022	0.21
	HIIT	20.66 ± 1.69	24.57 ± 1.93	3.91 ± 0.68*	<0.01	2.53; 5.29			
	CG	22.78 ± 1.63	24.04 ± 1.86	1.25 ± 0.65	0.06	−0.08; 2.58			
Long jump (cm)	SIT	106.35 ± 9.06	109.58 ± 11.93	3.23 ± 7.71	0.68	−12.46; 18.92	0.286	0.753	0.02
	HIIT	106.57 ± 7.54	117.23 ± 9.92	10.66 ± 6.42	0.11	−2.39; 23.71			
	CG	107.26 ± 7.26	113.68 ± 9.56	6.42 ± 6.18	0.31	−6.16; 19.00			

BMI, body mass index; BP, blood pressure; Chol, cholesterol; DBP, diastolic blood pressure; FM, fat mass; HDL, high density lipoprotein; LDL, low density lipoprotein; PR, pulse rate; SBP, systolic blood pressure; Trig, triglycerides; #Tendence to significance from corresponding CG value at the *p* < 0.07 level; *Significantly different from corresponding CG value at the *p* < 0.05 level; ** Significantly different from corresponding CG value at the *p* < 0.01 level.

**Table 3:** Differences between groups in the change pre-post intervention and interaction group*time adjusted by sex.

Outcome	Group	Adjusted by sex			Group * time interaction		
		Difference post-pre (M ± ED)	*p*	95% CI (Mpost-Mpre)	*F*	Sig	ES
BMI (kg/m2)	SIT	−0.29 ± 0.24	0.23	−0.78; 0.19	0.07	0.94	0.01
	HIIT	−0.18 ± 0.21	0.41	−0.61; 0.25			
	CG	−0.20 ± 0.26	0.44	−0.73; 0.32			
FM (%)	SIT	−2.42 ± 0.70	<0.01	−3.84; −1.00	4.03	0.03	0.19
	HIIT	−3.82 ± 0.62	<0.01	−5.07; −2.57			
	CG	−1.03 ± 0.75	0.18	−2.55; 0.50			
Lean mass (kg)	SIT	1.17 ± 0.43	0.01	0.29; 2.04	4.41	0.02	0.20
	HIIT	2.52 ± 0.38	<0.01	1.75; 3.30			
	CG	0.91 ± 0.46	0.06	−0.03; 1.85			
Trunk FM (kg)	SIT	−0.58 ± 0.17	<0.01	−0.93; −0.23	0.70	0.51	0.04
	HIIT	−0.50 ± 0.15	<0.01	−0.80; −0.19			
	CG	−0.29 ± 0.18	0.13	−0.66; 0.09			
BP (mmHg)	SIT	−1.11 ± 4.13	0.79	−9.49; 7.26	2.55	0.09	0.12
	HIIT	−12.76 ± 3.59	<0.01	−20.04; −5.47			
	CG	−3.96 ± 4.17	0.35	−12.43; 4.50			
SBP (mmHg)	SIT	0.06 ± 5.49	0.99	−11.08; 11.19	1.59	0.22	0.08
	HIIT	−12.75 ± 4.78	0.01	−22.44; −3.06			
	CG	−5.47 ± 5.55	0.33	−16.73; 5.78			
DBP (mmHg)	SIT	−1.70 ± 4.43	0.70	−10.67; 7.28	2.18	0.13	0.11
	HIIT	−12.76 ± 3.85	<0.01	−20.56; −4.95			
	CG	−3.21 ± 4.47	0.48	−12.28; 5.86			
PR (bpm)	SIT	−4.24 ± 3.04	0.17	−10.40; 1.92	5.29	0.01	0.23
	HIIT	−11.98 ± 2.64	<0.01	−17.33; −6.62			
	CG	1.04 ± 3.07	0.74	−5.18; 7.26			
Chol (mg/dL)	SIT	−5.35 ± 3.05	0.10	−11.81; 1.11	1.34	0.26	0.08
	HIIT	−0.49 ± 2.89	0.87	−6.61; 5.64			
LDL (mg/dL)	SIT	−0.34 ± 3.88	0.93	−8.57; 7.89	1.54	0.23	0.09
	HIIT	−6.99 ± 3.68	0.08	−14.80; 0.82			
HDL (mg/dL)	SIT	−0.43 ± 2.46	0.86	−5.64; 4.78	0.09	0.77	0.01
	HIIT	0.59 ± 2.33	0.80	−4.36; 5.53			
Trig (mg/dL)	SIT	−24.44 ± 19.07	0.22	−64.86; 15.99	4.30	0.05	0.21
	HIIT	30.09 ± 18.09	0.12	−8.25; 68.44			
LipidNonHDL (mg/dL)	SIT	−5.14 ± 3.06	0.11	−11.63; 1.35	0.93	0.35	0.05
	HIIT	−1.07 ± 2.90	0.72	−7.23; 5.08			
Chol /HDL (mg/dL)	SIT	−0.06 ± 0.10	0.57	−0.26; 0.15	0.11	0.75	0.01
	HIIT	−0.10 ± 0.09	0.29	−0.29; 0.09			
Glucose (mg/dL)	SIT	−8.51 ± 5.50	0.14	−20.18; 3.16	0.09	0.77	0.01
	HIIT	−6.24 ± 5.22	0.25	−17.31; 4.83			
Vo2máx (mL/kg/min)	SIT	0.63 ± 0.67	0.35	−0.73; 1.99	2.94	0.07	0.16
	HIIT	1.85 ± 0.52	<0.01	0.78; 2.92			
	CG	0.12 ± 0.50	0.82	−0.91; 1.14			
Handgrip (kg)	SIT	3.34 ± 0.86	<0.01	1.60; 5.08	4.27	0.02	0.22
	HIIT	3.85 ± 0.67	<0.01	2.48; 5.22			
	CG	1.25 ± 0.64	0.06	−0.06; 2.57			
Long jump (cm)	SIT	13.69 ± 6.40	0.04	0.64; 26.74	0.43	0.65	0.03
	HIIT	10.40 ± 5.02	0.05	0.17; 20.63			
	CG	6.42 ± 4.83	0.19	−3.42; 16.26			

BMI: body mass index; BP: blood pressure; Chol: cholesterol; DBP: diastolic blood pressure; FM: fat mass; HDL: high density lipoprotein; LDL: low density lipoprotein; PR: pulse rate; SBP: systolic blood pressure; Trig: triglycerides; #Tendence to significance from corresponding CG value at the *p* < 0.07 level; *Significantly different from corresponding CG value at the *p* < 0.05 level; ** Significantly different from corresponding CG value at the *p* < 0.01 level.

When comparing the pre-post-test changes between groups (HIIT, SIT and CG), the univariate analysis of the mean difference indicated a significant difference between HIIT and CG in the pre-post-test change in body fat percentage (%), lean mass (kg), heart rate and handgrip strength (kg). Which indicates that the group that received the HIIT program showed significantly different changes than the CG. Although not significant, males showed a greater improvement than females in Vo2max and long jump (Vo2max: SIT; *p* = 0.730; HIIT; *p* = 0.055/ Long jump: SIT; *p* = 0.905; HIIT = 0.354).

## 4 Discussion

This study evaluate the effects of HIIT and SIT programmes on body composition, blood pressure, lipid panel, glucose, cardiorespiratory fitness, and strength of adolescents and to compare the effect between those different protocols. As the present, previous studies have pointed to the ability of HIIT training to induce changes in fat percentage and trunk fat mass and increase lean mass, even over and above the changes induced by aerobic work or training based on physical activity recommendations from the World Health Organization (7, 8). Other studies have suggested that HIIT training, even when working with self-loading, as was the case in the present research, could increase lean body mass (9). This would be directly due to an increase in muscle mass because of the high tensile stress on skeletal muscle adaptation that occurs during HIIT, generating stimuli similar to those of resistance training (41). However, these previous studies had not analyzed which HIIT program would be most effective. One novelty of the present study is that it seems that the results on body composition are optimized when each HIIT series is maintained longer, even if it is at the cost of moderate intensity in comparison with vigorous intensity and with longer rests, with similar training volume.

Differences in the fat component could be explained by the longer periods of exercise at lower intensity may lead to a greater use of fat oxidation as a source of energy, while high-intensity exercise relies almost exclusively on carbohydrates as the main source of energy (42). Conversely, during HIIT intervals shorter than 30 s, energy demands are primarily met by the stored energy sources in muscle cells, such as adenosine triphosphate (ATP) and creatine phosphate (43). The observed advantages of this protocol in lean mass may be attributed to the dose–response relationship between muscle hypertrophy and training volume. Higher training volumes can lead to greater gains in muscle mass, with muscle failure promoted through the use of low-load as autoloads (44). Based on these findings, HIIT training at moderate intensity may be a promising approach for optimizing healthy changes in body composition in adolescents.

On the other hand, previous studies have shown that HIIT is an effective method of generating strength gains in schoolchildren after relatively short periods of time (45). The initial adaptations produced by strength and resistance training occur at a neural level, and it may take around 8 weeks to see changes at a structural level (46). However, the differences between the CG and HIIT in the handgrip were only significant, with no differences for either variable in relation to SIT. It could be hypothesized that less intense but more moderate HIIT over time generates faster adaptations in strength in adolescents. Previous studies have already suggested that strength adaptations may be specific to the training stimulus, following a comparison of three different resistance training programs (47).

However, although HIIT has been pointed out in previous research as a more effective method for improving VO2max than moderate-intensity continuous training, and many studies have focused on the changes it induces on this parameter (8), only HIIT maintaining each set at 120 s, showed significant changes in VO2max. To our knowledge, no previous studies have compared the effects of different HIIT protocols on cardiorespiratory fitness in adolescents. However, most studies suggest that there may be an effect of this type of training on VO2max from the fourth week of intervention (8). The absence of changes in SIT could be attributed to the difficulty of inactive adolescents complying with training based on vigorous physical activity, which usually yields no results. Another possible explanation could be that SIT intervals shorter than 30 s are covered energetically with the muscle cells’ oxygen stored in myoglobin, which helps sustain the exercise intensity without relying heavily on external sources of oxygen (48). Although the present study suggests the ability of HIIT to effect changes in cardiorespiratory fitness in adolescents, the study presents a HIIT structure that could be more effective for use in the school setting among adolescents. Therefore, this type of training could generate lower cardiovascular responses. Despite these promising results, questions remain.

Another relevant finding of the present study was that performing HIIT resulted in changes in the blood parameters of schoolchildren, whereas no changes were observed when the adolescents performed SIT. Previous studies have shown promising reductions in cardiometabolic risk factors using a HIIT program in adolescents, with improvements like those found with other exercise programs of longer duration such as steady-state exercise or resistance training (7). Another relevant finding of the present study was that performing HIIT resulted in changes in the blood parameters of schoolchildren, whereas no changes were observed when the adolescents performed SIT. Previous studies have shown promising reductions in cardiometabolic risk factors using a HIIT program in adolescents, with improvements similar to those found with other exercise programs of longer duration such as steady-state exercise or resistance training (49), or the main metabolic pathway demanded with the different HIIT protocols (42, 43), and further research is needed to investigate this issue.

In addition, our results indicate that males who trained with HIIT showed greater improvements in VO2max and standing long jump than females, although there were no significant differences in the improvements found according to sex. It is important to note that in the present investigation, the sample consisted of adolescents, who were around the age of peak altitude speed (APHV) (50). During this stage, in the case of males, testosterone increases up to 30 times the values measured before the growth spurt, which would explain the great improvement shown by males in fitness, while in females hormonal changes also occur during this period but much more gradually and to a lesser extent than in the male population (51).

Cardiovascular diseases are the leading cause of death in developed countries (1). To alleviate this pandemic, it is necessary to adopt healthy lifestyle habits from an early age, particularly by maintaining an active lifestyle (6, 52). The educational environment provides an ideal context for promoting such habits (53), and HIIT is a great alternative due to its high time/effect ratio. However, until now, it had not been analyzed which dose of HIIT could provide the highest stimulus/response ratio among adolescents (7–9).

The present study found that the implementation of a HIIT protocol within high school Physical Education sessions generated adaptations such as improved fitness condition, changes in body composition, and improvements in blood parameters and blood pressure. These adaptations appear to be protective against cardiovascular disease (5). In contrast, the group of adolescents who performed SIT, did not experience as many benefits, despite the similar duration of the program and stimulus time.

### 4.1 Limitations

The first limitation is the size of the sample and the inclusion of only one secondary school. Despite these limitations, this is the first study to analyze the effects of different HIIT protocols in the school setting on adolescents, and clear differences in the effects generated depending on the protocol used were found. Another strength of the present study is the large number of variables related to objectively measured cardiovascular parameters.

## 5 Conclusion

These results suggest that the implementation of a HIIT protocol within high school Physical Education sessions, maintained for 8 weeks, at a rate of 3 sets of 2 min of work (80–85% RHR)/2 min of rest (50–55% RHR) (duration: 12 min) generated adaptations such as improved fitness condition, changes in body composition, and improvements in blood parameters and blood pressure. These adaptations appear to be protective against cardiovascular disease (5). In contrast, the group of adolescents who performed SIT, shorter but more intense sets (6 sets of 60 s of work (90–95% resting heart rate (RHR))/60 s of rest (50–55% RHR)) did not experience as many benefits, despite the similar duration of the program and stimulus time. Therefore, physical education teachers and coaches should consider prescribing HIIT instead of SIT when the objective is the improvement of improved fitness condition, changes in body composition, and improvements in blood parameters and blood pressure. These findings represent great knowledge for public health and policies, providing greater understanding of the effects that physical exercise, specifically high-intensity interval training, has on adolescents. Considering the short development time of Physical Education sessions in the school context, this could be a valuable solution for the prevention of cardiometabolic diseases.

## Data availability statement

The raw data supporting the conclusions of this article will be made available by the authors, without undue reservation.

## Ethics statement

The studies involving humans were approved by the Institutional Ethics Committee of the San Antonio de Murcia Catholic University (code: CE061914). The studies were conducted in accordance with the local legislation and institutional requirements. Written informed consent for participation in this study was provided by the participants’ legal guardians/next of kin. Written informed consent was obtained from the individual(s), and minor(s)’ legal guardian/next of kin, for the publication of any potentially identifiable images or data included in this article.

## Author contributions

NG-G: Conceptualization, Data curation, Formal analysis, Funding acquisition, Investigation, Methodology, Project administration, Resources, Software, Supervision, Validation, Visualization, Writing – original draft, Writing – review & editing. JL-G: Formal analysis, Investigation, Resources, Visualization, Writing – original draft, Writing – review & editing. AE-G: Data curation, Investigation, Resources, Writing – original draft, Writing – review & editing. LA-C: Data curation, Investigation, Writing – original draft, Writing – review & editing. AM-O: Investigation, Writing – original draft, Writing – review & editing. RV-C: Investigation, Resources, Software, Validation, Visualization, Writing – original draft, Writing – review & editing.
